# Longitudinal progesterone profiles in baleen from female North Atlantic right whales (*Eubalaena glacialis*) match known calving history

**DOI:** 10.1093/conphys/cow014

**Published:** 2016-05-11

**Authors:** Kathleen E. Hunt, Nadine S. Lysiak, Michael J. Moore, Rosalind M. Rolland

**Affiliations:** 1 Research Department, New England Aquarium, Central Wharf, Boston, MA 02110, USA; 2 Woods Hole Oceanographic Institution, Woods Hole, MA 02543, USA

**Keywords:** Baleen, Cetacea, marine mammals, pregnancy, progesterone, reproduction

## Abstract

Reproduction of mysticete whales is difficult to monitor, and basic parameters, such as pregnancy rate and inter-calving interval, remain unknown for many populations. We hypothesized that baleen plates (keratinous strips that grow downward from the palate of mysticete whales) might record previous pregnancies, in the form of high-progesterone regions in the sections of baleen that grew while the whale was pregnant. To test this hypothesis, longitudinal baleen progesterone profiles from two adult female North Atlantic right whales (*Eubalaena glacialis*) that died as a result of ship strike were compared with dates of known pregnancies inferred from calf sightings and post-mortem data. We sampled a full-length baleen plate from each female at 4 cm intervals from base (newest baleen) to tip (oldest baleen), each interval representing ∼60 days of baleen growth, with high-progesterone areas then sampled at 2 or 1 cm intervals. Pulverized baleen powder was assayed for progesterone using enzyme immunoassay. The date of growth of each sampling location on the baleen plate was estimated based on the distance from the base of the plate and baleen growth rates derived from annual cycles of stable isotope ratios. Baleen progesterone profiles from both whales showed dramatic elevations (two orders of magnitude higher than baseline) in areas corresponding to known pregnancies. Baleen hormone analysis shows great potential for estimation of recent reproductive history, inter-calving interval and general reproductive biology in this species and, possibly, in other mysticete whales.

## Abstract (precis)

We compared known calving events to longitudinal baleen progesterone profiles from baleen plates of two stranded adult female North Atlantic right whales. Reproductive history of the previous ten years was recorded accurately in baleen, with pronounced elevations in progesterone in regions of baleen grown during known pregnancies.

## Introduction

Few methods exist for the study of reproductive endocrinology of the mysticete (baleen-bearing) whales. Improved understanding of pregnancies and inter-calving intervals in particular would be useful for population modelling and management of threatened and endangered populations. However, traditional endocrinological methods for monitoring pregnancies involve repeated blood sampling from individuals over many months or years, typically focusing on the steroid hormone progesterone, which is highly elevated in mammals during pregnancy (Bentley, 1998; Pukazhenthi and Wildt, 2004). In terrestrial wildlife, longitudinal monitoring of progesterone over time is commonly used to assess female fecundity, determine inter-birth intervals, identify cases of fetal or offspring loss and monitor oestrous cycling (Pukazhenthi and Wildt, 2004). This approach has been unfeasible in large whales because no method exists for live capture and release of unharmed individuals, and thus repeated blood samples cannot be collected (Hunt *et al.*, 2013). Valuable information has been gleaned from post-mortem examination of stranded specimens (Moore *et al.*, 2005, 2007), from resightings of known individuals (e.g. calf sightings combined with long-term photo-identification databases of well-studied populations; Hamilton *et al.*, 2007) and from endocrine analyses of alternative sample types (faeces, blubber and respiratory vapour; Rolland *et al.*, 2005; Hunt *et al.*, 2013; Kellar *et al.*, 2013), but repeated sightings or samples from the same female throughout a single pregnancy are rare. One encouraging recent approach involves analysis of hormones deposited in concentric layers of cerumen in whale earplugs; this method has the advantage of capturing the whale's entire lifetime, but is limited in that earplugs are not produced in all mysticetes, are difficult to excise, decompose post-mortem, and the temporal resolution of the tissue appears limited to 6 month intervals (Trumble *et al.*, 2013).

**Figure 1:**
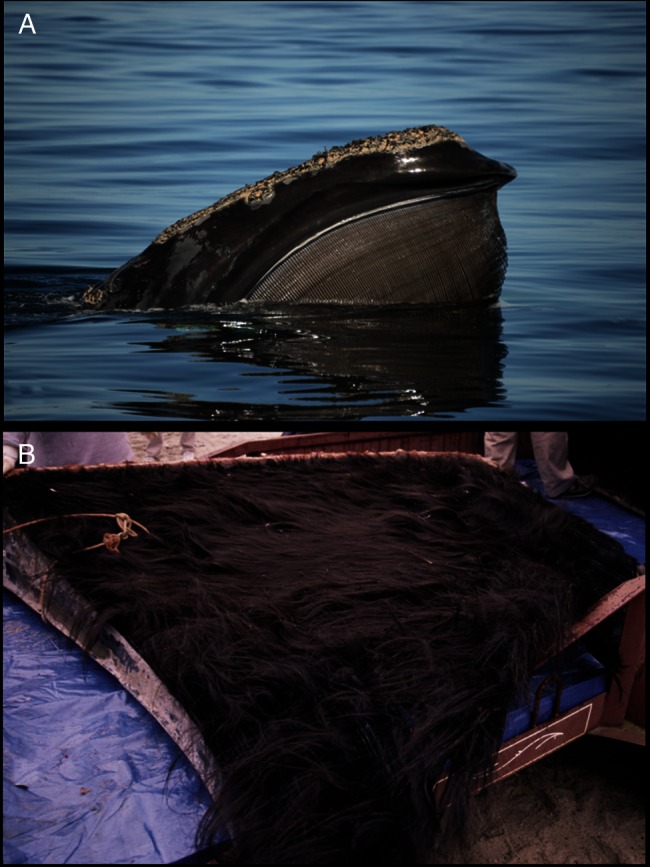
Baleen of North Atlantic right whales (NARW). (**A**) Rostrum of a living NARW, displaying edges of parallel baleen plates as used during filter-feeding of small prey near the water surface. (Photograph: Burgess/New England Aquarium, NMFS permit #14233.) (**B**) Excised baleen rack, showing the filament ‘fringe’ on the medial surface of baleen plates from an adult female NARW (Female 2 of this study). (Photograph: Moore/Woods Hole Oceanographic Institution NOAA Permit 932-1905-00/MA009526.)

Baleen, a tissue type produced by all mysticete whales, is a potential alternative. Baleen consists of flexible, long, thin ‘plates’ (vertical strips) suspended in parallel from the palate of the upper jaw (Fig. 1A), each plate consisting of long, hair-like filaments of α-keratin embedded within a hard matrix of amorphous keratin and calcium salts (St Aubin *et al.*, 1984; Young *et al.*, 2015). Racks of baleen plates on the left and right side of the mouth function as a filter-feeding apparatus, with the filter bed consisting of a fringe of free filaments emanating from the medial aspect of the hard matrix (Fig. 1B; Young, 2012). Stable isotope data indicate that in adult baleen whales, each baleen plate grows slowly and continuously from the base (upper or proximal end) and constantly wears away at the tip (lower or distal end; Best and Schell, 1996; Lee *et al.*, 2005; Lubetkin *et al.*, 2008; Lysiak, 2009; Matthews and Ferguson, 2015). In species with long baleen, such as bowhead whales (*Balaena mysticetus*) and right whales (*Eubalaena* spp.), a single baleen plate can represent a decade or more of continuous growth (Best and Schell, 1996; Lee *et al.*, 2005; Lubetkin *et al.*, 2008; Matthews and Ferguson, 2015). Baleen is routinely collected from stranded specimens and does not decompose post-mortem.

The filaments in baleen are similar in structure and composition to hair of other mammals (St Aubin *et al.*, 1984; Pfeiffer, 1992). It is now well established that mammalian hair contains steroid hormones that are thought to be deposited in the hair shaft while the hair is growing (Yang *et al.*, 1998; D'Anna-Hernandez *et al.*, 2011; Meyer and Novak, 2012). Thus, a single long hair can contain a continuous endocrine record of the entire time period during which that hair was grown. The close similarity of mammalian hair to baleen filaments (Fig. 1B) suggested that steroids might also be deposited in baleen. We recently demonstrated that fresh (recently grown) baleen of bowhead whales contains detectable progesterone and cortisol (Hunt *et al.*, 2014). We hypothesized, therefore, that full-length baleen plates might contain continuous endocrine records that could span the duration of multiple pregnancies, potentially enabling the first retrospective hormonal assessment of pregnancies and inter-calving intervals from individual mysticete whales. The accuracy of such an approach must be tested by comparison of progesterone profiles to independently confirmed calving history, but baleen plates from females of known calving history are extremely rare. Additionally, it was unknown whether progesterone would remain detectable throughout the full length of the baleen plate, even in older baleen that has been exposed to seawater for many years.

To answer these questions, we compared longitudinal baleen progesterone concentrations (from sequential locations along baleen plates) with the known calving history of two individual female North Atlantic right whales (*Eubalaena glacialis*, NARW). Our primary question was whether baleen progesterone is significantly elevated in areas of baleen that were grown during pregnancy (i.e. the year before first sighting of a calf; whale definitely pregnant) compared with the year after calving (i.e. whale was definitely not pregnant; NARW lactate for 1 year and have never been documented to give birth in two successive years; Hamilton *et al*., 1995; Kraus *et al.*, 2007). A secondary question was whether either female showed indications of previously undetected pregnancies (e.g. pregnancy loss or loss of an undetected calf) during the inter-calving interval after the lactation year.

## Materials and methods

### Study animals

We obtained baleen plates at post-mortem examination from two NARW females with detailed reproductive history, referred to here as Female 1 (Eg #1004, ‘Stumpy’) and Female 2 (Eg #1014, ‘Staccato’). The longest baleen plate (‘full-length’ plate) from each individual was collected. The NARW, a critically endangered species, was chosen as a study species because individual reproductive histories are known for many individuals, owing to an extensive photo-identification catalogue based on individually unique patterns of head callosities in this species (NARW Identification and Sightings Database; Hamilton *et al.*, 2007; North Atlantic Right Whale Consortium, 2015), along with an intensive population monitoring effort that includes annual surveys of calving and feeding grounds (Brown *et al.*, 2007). In the decade before their deaths, both females had been sighted repeatedly in seasonal foraging and calving habitats, and each had two documented pregnancies.

Complete records of sightings for these two individuals are available online from the North Atlantic Right Whale Catalog (www.rwcatalog.neaq.org; North Atlantic Right Whale Consortium, 2015). Female 1 was first sighted in 1975 and was found dead on 7 February 2004, with injuries consistent with ship strike. She was sighted on the calving grounds with a young calf in December 1996 and was found at post-mortem examination to be pregnant with a full-term fetus (North Atlantic Right Whale Consortium, 2015). Female 2 was first sighted in 1974 and was found dead on 20 April 1999, also with injuries consistent with ship strike; she was sighted on the calving ground with young calves in December 1990 and December 1996 (Moore *et al.*, 2005; North Atlantic Right Whale Consortium, 2015). Dates of calf sightings (or, in the case of Female 1, size of fetus at post-mortem examination) were used to predict locations of high-progesterone areas in the baleen, based on an estimated gestation length of 12–13 months (Best, 1994) and an average baleen growth rate for adult female NARW (derived from stable isotope data; see “Stable Isotope Analysis,” below).

### Cleaning of plates

Connective tissue and gingiva were removed from the baleen plates after collection. At the time of post-mortem examination (prior to this study), the post-mortem coating of exuded, decomposing internal body lipids on the baleen of Female 1 was removed with a commercial shampoo (Clairol ‘Herbal Essences’; main detergent ingredients include sodium lauryl sulfate and sodium laureth sulfate). This treatment was not required for Female 2. Plates were then archived at room temperature at the Woods Hole Oceanographic Institution (Woods Hole, MA, USA) for potential future study. The plates were transferred to the New England Aquarium (Boston, MA, USA) in 2014, where the plates were cleaned again by wiping three times with laboratory tissues wetted with 70% ethanol, following established protocols from hair hormone literature (Davenport *et al.*, 2006; Ashley *et al.*, 2011; Bechshøft *et al.*, 2011). After final cleaning, the baleen was handled only with gloved hands to avoid contamination with hormones present in human skin oils. The plates were analysed for hormone content in early 2015.

### Stable isotope analysis

**Figure 2:**
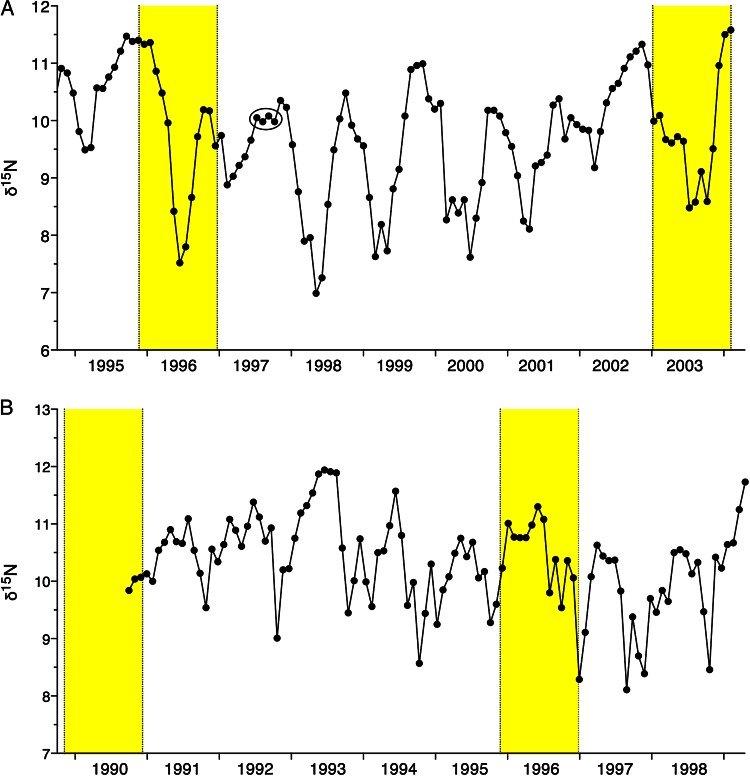
Baleen nitrogen (δ^15^N) stable isotope records for North Atlantic right whales. (**A**) Female 1 (Eg #1004 ‘Stumpy’, *n* = 115). (**B**) Female 2 (Eg#1014 ‘Staccato’, *n* = 105). Yellow bars indicate predicted high-progesterone regions based on calf sightings, post-mortem examination data and the estimated 12–13 month gestation length for this species. Circle in panel (A) is an example of a time period with multiple sightings of Female 1 in the same, isotopically distinct, geographical area (Bay of Fundy sightings in June, July, August and September 1997) corresponding to points on the baleen that had similar stable isotope ratios.

The baleen growth rate (BGR) was estimated from seasonal patterns in stable isotopes along full-length baleen plates. In a separate study, carbon (δ^13^C) and nitrogen (δ^15^N) stable isotope ratios were analysed for multiple female adult NARW (including the two individuals in this study, although sampling different baleen plates), producing an estimated baleen growth rate of ∼1 cm of baleen per 15 days (Lysiak, 2009). Briefly, δ^13^C and δ^15^N in longitudinally sampled baleen contain oscillating patterns that are believed to be annual, formed when whales feed on zooplankton in isotopically distinct food webs over the course of a year (Best and Schell, 1996; Lee *et al.*, 2005; Matthews and Ferguson, 2015). In the northwest Atlantic, the δ^15^N gradient that NARW traverse during seasonal migrations provides the clearer signal of the two isotopes (Lysiak, 2009; McMahon *et al.*, 2013). Annual patterns in baleen δ^15^N were used to estimate a population average BGR for adult female NARW of ∼1 cm of baleen per 15 days. These estimates of BGR were validated by cross-referencing stable isotope measurements of zooplankton prey collected in known NARW feeding habitats, as well as the sighting records from individual whales (Wetmore, 2001; Lysiak, 2009; North Atlantic Right Whale Consortium, 2015). The BGR for both individuals in the present study was cross-checked against the population average (1 cm per 15 days) by measuring the distance between annual peaks in δ^13^C and δ^15^N baleen profiles for each individual whale (Fig. 2; for clarity, only δ^15^N profiles are shown) and multiplying those counts by the 2 cm sampling interval; see Lysiak (2009) for full details. Individual sightings records were also inspected for concordance of sightings data with stable isotope data and estimated BGR. For example, Female 1 was sighted in the Bay of Fundy habitat with her 1997 calf on several occasions in June, July, August and September 1997. These sightings, corresponding temporally to the points circled in Fig. 2A, provide an ‘anchor point’ that lends support to the estimated BGR, because those points have nearly identical stable isotope ratios, consistent with a long residence within a single (isotopically distinct) habitat area. The previously reported BGR of 1 cm per 15 days for adult female NARW (Lysiak, 2009) produced the best fit to the available stable isotope data and sightings records for these two individual whales, so this BGR was used to estimate the date of growth of each point on the baleen.

### Measurement of baleen plates and estimation of date of growth

A tape measure was permanently affixed to each baleen plate (on the posterior face of the plate on the labial edge, with the tape following the natural curve of the plate), and 2 cm intervals were marked along the entire length of this tape, with the proximal end of the base of the plate designated as the ‘zero-cm’ point. This tape measure remained attached to the baleen plate for the duration of the study. The baleen at the zero-cm point was conservatively assigned an estimated growth date of the day before the whale was found dead; note that if whales died more than 1 day before the carcass was first seen, the apparent time line will be shifted accordingly (e.g. estimated dates of baleen growth will appear to be slightly later in time than they really were). All other points along the baleen plate were assigned an estimated date of growth based on distance from this zero-cm point and the BGR described above (1 cm baleen grown per 15 days).

### Pulverization of baleen

Based on a protocol tested in bowhead baleen (Hunt *et al.*, 2014), baleen samples were pulverized with a hand-held electric rotary grinder (Dremel model 395 type 5) fitted with a tungsten ball-tip and flexible extension. At 4 cm intervals, from base to tip, we drilled a short (∼4 cm) transverse groove across the posterior face of the plate, starting at the labial edge of the plate and following visible transverse lines in the baleen (thought to be growth lines). Powdered baleen was collected on a piece of weigh paper below the plate. Considerable care was taken to avoid cross-contamination by shielding other regions of the plate during the drilling process and by thoroughly recleaning the baleen, all equipment and the work area with 70% ethanol between samples, with multiple changes of gloves. The baleen plate from Female 2 had a layer of dried white gum tissue adherent to the proximal 24 cm of the plate; this gum tissue was removed, and only the black baleen underneath was sampled. Small amounts of gum tissue may have contributed to the first three samples owing to thinness of the baleen in this region, but the contribution of gum tissue is believed to be minimal. The baleen plate from Female 1 had no visible gum tissue.

### Weighing of powder and extraction of hormones

Optimal extraction techniques for baleen powder are not yet known. Based on hair cortisol and feather corticosterone protocols (Davenport *et al.*, 2006; Bortolotti *et al.*, 2008; Ashley *et al.*, 2011; Bechshøft *et al.*, 2011; Lattin *et al.*, 2011) and a previous pilot study in bowhead baleen (Hunt *et al.*, 2014), 100 mg of well-mixed baleen powder was combined with 4.00 ml of 100% methanol in a borosilicate glass test tube, vortexed for 20 h at room temperature, centrifuged for 15 min at 4000***g***, and the supernatant transferred to a dry-down tube. The pellet was then rinsed twice, each rinse consisting of 1.0 ml methanol added to the pellet, vortexing for 1 min, centrifuging for 5 min at 4000***g***, and combining supernatants. The final combined supernatant (4 + 1 + 1 ml) was then dried down overnight at 45°C under air blow in a Reacti-therm™ dry-block sample evaporator. Near the end of drying, a final 0.5 ml of 100% methanol was added to rinse down any hormone that had dried high on the wall of the tube, and dried again. Finally, samples were reconstituted in 0.50 ml assay buffer (‘progesterone assay buffer’; Arbor Assays, Ann Arbor, MI, USA), vortexed for 1 min, sonicated for 1 min, transferred to cryovials, spun in a minifuge for 10 s, decanted to a new cryovial to remove any remaining baleen particulates, and frozen at −20°C. Extracts were assayed within a month.

### Hormone assays

All samples were assayed with a commercial progesterone enzyme immunoassay kit (catalogue #K025-H1; Arbor Assays). This assay was selected based on previous successful use in a pilot trial with baleen extracts of bowhead whales (Hunt *et al.*, 2014) and its use of an antibody widely employed for unusual sample types in mammalian wildlife (antibody ‘CL425’). The assay has seven standards spanning 50–3200 pg/ml. The manufacturer's reported sensitivity limit is 47.9 pg/ml, intra-assay precision is <5.1%, and inter-assay precision is <7.0%; for further details, see Hunt *et al.* (2014). Assay validations employed standard parallelism and accuracy tests (Grotjan and Keel, 1996). For the parallelism test, a serially diluted pool of NARW baleen extract (produced by combining equal volumes of extract from both females) was assayed, and the slope of percentage bound vs. logarithm of relative dose was compared with the standard curve. The two curves were parallel (linear portions of curves compared; *F* test, *F*_1,6 _= 0.1038, *P *= 0.7582), indicating that the assay can detect progesterone in NARW baleen extract across a range of dilutions. Accuracy was next tested by assaying progesterone standards spiked with equal volumes of a 1:4 dilution of pooled baleen extract. The resulting graph of observed vs. expected dose was linear (*r*^2^ = 0.9968; runs test, no significant deviation from linearity), with a slope within the desired range of 0.7–1.3 (slope = 0.74), indicating that high-dose samples are correctly discriminated from low-dose samples. Based on pilot data, all samples were diluted 4-fold in assay buffer (‘1:4’) for the initial assay. Samples and standards were assayed in duplicate, and non-specific binding and blank wells were assayed in quadruplicate. Samples were assayed blind, with samples from each whale grouped in a single assay. Any samples with extremely high progesterone (percentage bound <5%) were then diluted to 1:40 or 1:400 and re-assayed so as to bring them closer to 50% bound on the standard curve, the area of greatest assay precision. Any samples that fell outside 10–90% bound on the standard curve, that had >10% coefficient of variation between the two duplicates or that had anomalous results compared with neighbouring samples were re-assayed. Final results were converted to nanograms of immunoreactive progesterone per gram of baleen powder.

### Resampling of high-progesterone regions

In each baleen plate, the initial round of assays identified certain ‘high-progesterone regions’ representing possible pregnancies. To investigate these areas more thoroughly, all high-progesterone regions were later resampled at 2 cm intervals, i.e. filling in the intermediate points between the 4 cm intervals originally sampled. Additionally, in each baleen plate the first third of the most recent pregnancy was selected for even finer sampling, at 1 cm intervals, in order to examine the beginning of an apparent pregnancy in each individual. Final sample sizes, including the ‘resampled’ areas, were 89 samples from Female 1 and 88 samples from Female 2.

## Results

**Figure 3:**
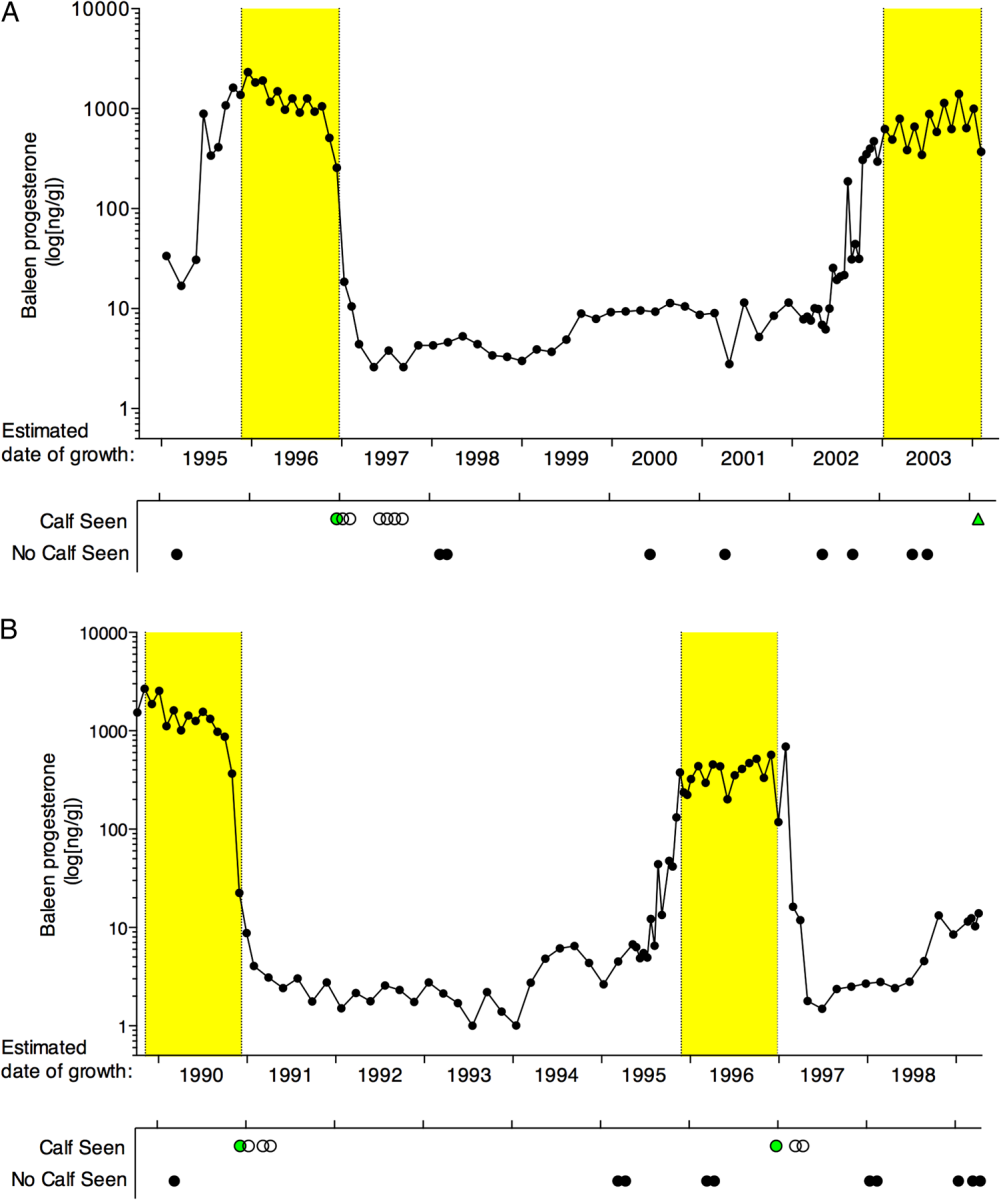
Predicted high progesterone (yellow bars), observed baleen progesterone profiles (black dots with lines) and sightings records of two North Atlantic right whale females. (**A**) Female 1 (Eg #1004, ‘Stumpy’, *n *= 89 samples). (**B**) Female 2 (Eg #1014, ‘Staccato’, *n* = 88 samples). The *x*-axis depicts estimated dates of growth of each point along the baleen plate, derived from the distance from the proximal-most point on the plate (newest baleen) and the estimated baleen growth rate (from stable isotope data). Monthly sightings records are shown below for each whale, with multiple sightings within each month summarized as follows: green circle, female sighted with neonatal calf on the calving grounds; open circle, female sighted with an older (non-neonatal) calf; black circle, female sighted without a calf; and green triangle, near-term fetus discovered at post-mortem examination (Female 1 only). Note the logarithmic scale of the *y*-axis.

Baleen progesterone profiles (Fig. 3) showed close correspondence with known calving history. Each baleen plate contained two regions with markedly elevated progesterone concentration (approximately two orders of magnitude above baseline), and the estimated dates of growth of these high-progesterone regions corresponded well to the two known pregnancies for each whale. Generally, progesterone increased from <10 to >100 ng/g during each known pregnancy, remained >100 ng/g for more than a year before a calf was sighted, and dropped to <10 ng/g within ∼1 month of the first sighting of a neonatal calf. Baleen progesterone remained <100 ng/g for the entire duration of the inter-calving interval until the next known pregnancy began.

## Discussion

Baleen of these female North Atlantic right whales contained a continuous, longitudinal time series of progesterone concentrations that accurately reflected the known calving history of the previous 10 years. In both whales, progesterone was elevated by one to two orders of magnitude in the year prior to birth of a calf (i.e. whale definitely pregnant) compared with the year after (i.e. whale definitely not pregnant). This ‘pregnancy signature’ in the baleen was remarkably pronounced, remaining easily detectable even at the distal tip of the baleen plate, a region that had been continuously submerged in seawater for nearly a decade and subsequently stored for over a decade at room temperature.

### Inter-calving intervals

Progesterone remained very low (<10 ng/g) for 2–3 years after birth of a calf. The low progesterone in the first year of the inter-calving interval (the lactation year; Hamilton *et al*., 1995) may reflect lactational suppression of ovarian activity, common in mammals (Bentley, 1998) but not documented before in mysticete whales. Progesterone then showed a small but distinct elevation in either the third (Female 1) or fourth (Female 2) year after birth of the calf, possibly signifying a resumption of ovarian activity. However, baleen progesterone was not elevated further for two (Female 1) or three (Female 2) more years.

In both females in this study, the two pregnancies were separated by an unusually long inter-calving interval of 6 years (Female 2) or 7 years (Female 1). Inter-calving intervals averaged 3.7 years for this species in the late 1980s and lengthened (for unknown reasons) to ∼5.8 years during the 1990s (Kraus *et al.*, 2007), the period represented by both baleen plates in this study. In such cases of lengthened inter-calving interval, it is typically unclear whether a female might have been pregnant during the lengthened inter-calving interval, with subsequent loss of the fetus or calf (or the calf having never been sighted) or whether the female simply did not become pregnant. In these two individuals, during the entire inter-calving interval between the two known pregnancies, baleen progesterone remained more than an order of magnitude lower than levels typical of known pregnancies. It therefore appears that neither female became pregnant during the lengthened inter-calving intervals, i.e. there are no obvious indications of pregnancy failure nor loss of an undetected calf. These data represent the first endocrinological information concerning whether the extended inter-calving intervals that are occasionally observed in this species represent failure to conceive vs*.* increased rate of miscarriage or calf mortality. Ascertaining the cause of reproductive failure in endangered species is a question of considerable interest; for example, conception failure, loss of pregnancies or high calf mortality can potentially be attributable to very different environmental and anthropogenic causes. In species with annual migrations, these differential causes of reproductive failure can also involve distinctly different seasons and geographical locations. Such information could prove valuable, in future studies, for determining appropriate management interventions.

### Gestation length

The high-progesterone regions spanned a longer stretch of baleen than anticipated, bracketing time periods longer than the expected 357–396 day (∼12–13 month) gestation length. Using a tentative assumption of relatively constant BGR across all months, and defining pregnancy regions conservatively as an uninterrupted series of samples that all have progesterone >100 ng/g, the two pregnancies of Female 1 span an apparent 540 days (1995–96 pregnancy) and 481 days (2003–04 pregnancy; this female had not yet given birth when killed). The 1989–90 pregnancy of Female 2 spanned a minimum of 391 days (this pregnancy is incompletely recorded owing to erosion of the tip of the baleen plate, and had very high progesterone even in the oldest point sampled), and her 1995–96 pregnancy spanned an apparent 451 days.

The apparent mismatch of duration of high progesterone to estimated gestation length could be a methodological artefact, owing to potential factors that include the following. First, the progesterone concentration of a given location along the baleen plate might not represent a single point in time. Little is known about the deposition of progesterone within baleen, nor about potential variation in deposition within and across the baleen plate; thus, the progesterone concentration at any given location might represent not a single point in time, but an average of a few weeks or even months. Second, BGR might vary seasonally or might vary during pregnancies. There was close correspondence between the estimated date of the reduction of progesterone with the known date of sighting of a neonatal calf, even for the older pregnancies; this indicates that our estimate of average BGR across multiple years is likely to be reasonably accurate for both whales. Nonetheless, there might be variation of BGR within years, i.e. seasonal variation in BGR and/or variation with reproductive state. Variation in the hair growth rate during pregnancy occurs in other mammals (LeBeau *et al.*, 2011); stable isotope data and sightings data put upper bounds on the degree of potential variation in BGR in these two individuals (see Materials and methods), but minor variations in BGR are possible. Variable BGR might also explain the variation in apparent gestation length between the different pregnancies. Third, oestrous cycling immediately before a pregnancy might affect the progesterone content of the baleen; i.e. the earliest part of apparent ‘pregnancies’ might in fact represent luteal phases of oestrous cycles. Oestrous cycle duration and characteristics for mysticetes are presently unknown, but the earliest third of the more recent pregnancies (sampled at 1 cm intervals) have some indications of possible cycling, e.g. brief and fluctuating elevations in baleen progesterone.

Finally, it is also possible that gestation length in NARW might be slightly longer than previously estimated. The 12–13 month gestation length widely cited for NARW is derived from estimates of the duration of the non-linear phase of fetal growth rate in the southern right whale, *Eubalaena australis* (Best, 1994). The NARW, a different (although closely related) species, might have a slightly different gestation length (Best, 1994; Cole *et al.*, 2013). However, we emphasize that at present it is unclear whether baleen hormone profiles have precise enough time resolution for accurate measurement of gestation length. Further studies will be necessary to characterize the time frame of hormone deposition within baleen plates, variation in hormone concentration across different points on the baleen, variation in BGR and the potential influence of oestrous cycling.

### Conclusions

These data represent the first detailed longitudinal progesterone profiles reported for mysticete whales and the first endocrine profiles encompassing complete pregnancies. Although data are as yet available from only two individuals, the very strong ‘pregnancy signal’ of elevated progesterone observed in both individuals, along with the good match in timing between elevated progesterone and known pregnancies, suggests that baleen progesterone profiles could be used to estimate inter-calving intervals in this species and, possibly, in other mysticetes. Our study also demonstrates that progesterone is easily detectable even in the very oldest baleen sampled (tip of the baleen plates, exposed to seawater for 9–10 years) and even after a decade or more of storage of the baleen at room temperature. This result is in good agreement with findings that steroid hormones show great longevity in mammalian hair, in some cases remaining detectable for centuries (Bechshøft *et al.*, 2012; Webb *et al.*, 2014). Although many validations remain to be done, particularly on the variation in baleen growth rate and the precision of time resolution, it is possible that contemporary samples as well as historical archives of baleen in museums, oceanographic institutions and archeological sites may contain information on pregnancy, inter-calving rate and other aspects of endocrinology that could enable improved understanding of patterns of reproduction in these difficult-to-study species.

## Funding

This work was supported by the Eppley Foundation for Research, the National Oceanographic and Atmospheric Administration Marine Mammal Health and Stranding Program and the Woods Hole Oceanographic Institution Ocean Life Institute.
